# Unconventional Use of LC3 by Coronaviruses through the Alleged Subversion of the ERAD Tuning Pathway

**DOI:** 10.3390/v3091610

**Published:** 2011-09-05

**Authors:** Fulvio Reggiori, Cornelis A.M. de Haan, Maurizio Molinari

**Affiliations:** 1 Department of Cell Biology and Institute of Biomembranes, University Medical Center Utrecht, Heidelberglaan 100, 3584 CX Utrecht, The Netherlands; 2 Virology Division, Department of Infectious Diseases and Immunology, Utrecht University, Yalelaan 1, 3584 CL Utrecht, The Netherlands; 3 Institute for Research in Biomedicine, Via Vela 6, 6500 Bellinzona, Switzerland; 4 Ecole Polytechnique Fédérale de Lausanne, School of Life Sciences, 1015 Lausanne, Switzerland

**Keywords:** coronaviruses, *nidoviruses*, double-membrane vesicles, MHV, SARS-coronavirus, autophagy, EDEMosomes, EDEM1, OS-9, ERAD, ER quality control

## Abstract

Pathogens of bacterial and viral origin hijack pathways operating in eukaryotic cells in many ways in order to gain access into the host, to establish themselves and to eventually produce their progeny. The detailed molecular characterization of the subversion mechanisms devised by pathogens to infect host cells is crucial to generate targets for therapeutic intervention. Here we review recent data indicating that coronaviruses probably co-opt membranous carriers derived from the endoplasmic reticulum, which contain proteins that regulate disposal of misfolded polypeptides, for their replication. In addition, we also present models describing potential mechanisms that coronaviruses could employ for this hijacking.

## 1. Introduction

Coronaviruses (CoV) are enveloped viruses with plus-strand RNA genomes belonging to the family *Coronaviridae,* which together with the *Roni-* and the *Arteriviridae*, form the order Nidovirales [1]. CoV infect a large range of birds and mammals, including humans, and their pathogenesis has been intensively studied since the 1960s. CoV generally cause respiratory and/or intestinal infections, although some may spread systemically. Human CoV (HCoV)-OC43 and HCoV-229E, for example, cause mild upper respiratory tract infections [2], although they are occasionally associated with severe pulmonary diseases in newborns and immuno-compromised people [3]. In spring 2003, a new human CoV became infamously notorious due to an outbreak in South East Asia and Canada [4,5]. The accused virus was rapidly identified as the SARS-CoV. Unlike HCoV-OC43 and -229E, the SARSCoV causes a severe respiratory disease [6], with nearly 10% mortality and it also spreads systemically [7]. Since 2003, two additional new human CoV have been characterized, *i.e.*, HCoV-NL63 and HCoV-HKU-1, which are also mainly associated with mild upper respiratory tract infections [8–10].

Several animal CoV are economically important pathogens [11]. For example, transmissible gastroenteritis virus (TGEV) causes diarrhea in pigs [12–14], feline infectious peritonitis virus (FIPV) leads to a fatal systemic disease in cats [15–17], the bovine coronavirus (BCoV) causes respiratory tract diseases and diarrhea in cattle [14,18] and the avian infectious bronchitis virus (IBV) is the etiological agent of severe respiratory tract and kidney diseases in chickens [14,19]. The mouse hepatitis virus (MHV) has been extensively used to study the replication and assembly of CoV in cell culture models as well as in whole animals and is thus considered as a prototype CoV [11,14].

## 2. CoV Life Cycle

CoV virions are spherical enveloped particles, with a diameter of 70–120 nm [20]. All CoV particles contain a common set of 4 structural proteins, *i.e.*, the spike (S), the membrane (M) and the envelope (E) transmembrane proteins, and the nucleocapsid (N) protein. The large genomic RNA is encapsidated by multiple copies of the N protein forming the helical nucleocapsid. The CoV envelope, which is composed of a lipid bilayer derived from host intracellular membranes, accommodates the 3 transmembrane proteins. The S protein forms the peplomers that radiate from the virion surface. The M protein is the most abundant protein in the virus particle while the E protein is only present in small amounts [21,22].

**Figure 1.**
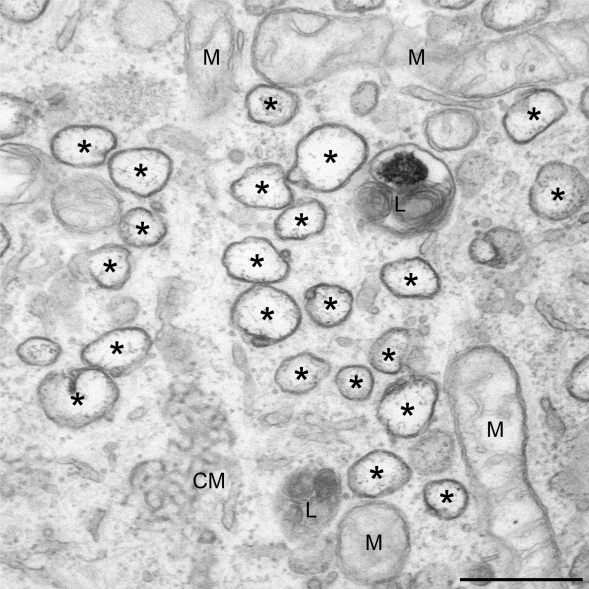
Ultrastructure of membrane-associated replicative structures induced by mouse hepatitis virus (MHV) in host cells. Mouse LR7 cells inoculated with the MHV-A59 strain were fixed at 10 h post-infection and processed for conventional EM. Double membrane vesicles (DMVs) are often found clustered together in close proximity of a small network of membranes, the CMs, which are morphologically distinct but have identical viral protein composition. The asterisks mark the DMVs. CM, convoluted membranes; M, mitochondria; L, lysosome. Size bar, 500 nm.

The first step in the CoV infection cycle is the association of the virion with the host cell surface through the binding of the S proteins to specific receptors. After endocytic uptake, conformational changes in the S protein result in fusion of the viral envelope with a limiting cellular membrane [23–27]. Upon virus-cell fusion, the virions disassemble and release their genomic RNA in the cytoplasm. The viral RNA is directly translated into two very long polypeptides of approximately 400 and 800 kDa, called pp1a and pp1b, respectively [11,28,29]. During and after their synthesis, pp1a and pp1b are cleaved by viral proteinase activities contained in their sequence and this processing leads to the generation of 16 non-structural proteins (nsp’s) [11,30]. These factors probably trigger the rearrangement of host cellular membranes, resulting in the formation of a reticulovesicular network of double membrane vesicles (DMVs) and convoluted membranes (Figure 1) and form replication-transcription complexes (RTCs) that are associated with the rearranged membranes [31–35].

The RTCs copy the genomic RNA either continuously into genome-length or discontinuously into various subgenome-length minus-strand templates. The minus strands are in turn used as templates for the synthesis of genomic and subgenomic mRNA [35,36]. These latter products comprise a nested set of overlapping species of mRNAs that extend for different lengths from a common 3′ terminus [35,36]. Replication and transcription of plus-strand RNA viruses result in the formation of double-stranded RNA (dsRNA) molecules that are generally assumed to function as intermediates in the RNA synthesis [36,38]. The exact location of viral RNA synthesis remains unknown at present. Although it has been shown that dsRNA accumulates in the interior of DMVs, the significance of this phenomenon remains unclear [31]. The dsRNA generated by viruses are potent inducers of antiviral interferon signaling pathways [39,40] and therefore its sequestration into DMVs could prevent the activation of these innate immune responses [31,38].

The structural proteins are synthesized from the subgenomic RNAs and the ones with transmembrane segments are subsequently inserted in the limiting membrane of the endoplasmic reticulum (ER) [41–43]. The S protein is found along the secretory pathway and at the plasma membrane. The M protein, in contrast, localizes predominantly in the Golgi compartments while the E protein is detected in the ER, in the Golgi, and in the ER-to-Golgi intermediate compartment (ERGIC). Nonetheless, these proteins and the helical nucleocapsids cooperatively assemble into virions via lateral interactions that induce the invagination and luminal pinching off of the limiting membrane of the ERGIC. The resulting luminal virions subsequently reach the extracellular environment following the conventional secretory pathway.

## 3. The ER Origin of the CoV-Induced DMVs

All plus-strand RNA viruses synthesize their viral RNA in association with extensive virus-induced rearrangements of specific intracellular membranes. Different plus-strand RNA viruses may target different membrane compartments, such as the ER, endosomes, mitochondria or chloroplasts, thereby giving rise to membrane invaginations, (clusters of) DMVs or single-membrane vesicles, membrane-bound vesicle packets, convoluted membranes and other structures or combinations thereof (for reviews see [38,44]). For a long time, the subcellular compartment from where the membranes composing the CoV-induced DMVs are derived has remained mysterious. The major difficulty in solving this issue has been the lack or the undetectable levels of marker proteins of subcellular organelles [33,45–47], the detection of which could have provided insight into the origin of these structures. Even so, several pieces of evidence has indicated that the ER is the most probable source for the lipid bilayers composing the CoV-induced DMVs. First, nsp3 and nsp4, two nonstructural proteins with transmembrane segments, which are very likely part of the RTCs, become N-glycosylated [48–51], a co-translational modification that occurs in the ER. Second, nsp4 localizes to the ER when ectopically expressed and moves to the DMVs upon viral infection [48]. Third, a block of the early transport steps of the secretory pathway inhibits CoV replication [46–48]. Fourth, ultrastructural studies where electron tomography was applied, demonstrated that the CoV-induced DMVs are interconnected via their outer membranes and are part of a membranous reticulovesicular network, which also includes convoluted membranes and is connected to the ER [31]. Fifth, Sec61α, which is a subunit of the ER translocon, redistributes upon SARS-CoV infection and localizes to the rearranged membranes [47]. Finally, the DMVs induced in SARS-CoV infected cells contain ribosomes on their outer membranes [31], although this has not been observed in MHV-infected cells [33,45].

Even though the ER is the likely (possible) source of the membranes composing the CoV-induced DMVs, the lack of ER, ERGIC or Golgi protein markers, as well as the absence of coatomer proteins at their limiting membranes [31,33,46,47] question a model in which CoV co-opt ER-derived secretory vesicles for DMVs formation. Based on available data, it seems more plausible that DMVs might result from extensive modifications of ER membranes [47] or of ER-derived vesicles that regulate ERAD tuning, *i.e.*, the export from the ER of EDEM1, OS-9 and other regulators of ER-associated degradation (ERAD) [45]. In the next sections, we will mainly review this latter scenario.

## 4. ERAD, ERAD Tuning and its Subversion by CoV

The ER is the site of maturation for secretory and membrane proteins in eukaryotic cells. Proteins that fail to attain the native structure must efficiently be removed from the ER lumen to protect cells from stress conditions eventually leading to cell death. Thus, besides molecular chaperones and folding enzymes that assist maturation of newly synthesized proteins, the ER also contains ERAD factors that recognize non-native proteins, extract them from the folding machineries and ensure their transport across the ER membrane for proteasomal degradation [52,53]. The intraluminal concentration of ERAD factors must be tightly regulated. When present in excess, ERAD factors might interfere with ongoing folding programs and trigger inappropriate degradation of not-yet-native folding intermediates. This might eventually compromise the ER capacity to efficiently produce functional polypeptides and may correlate, for example, with the enhancement of the metastatic potential of tumor cells caused by the inappropriate degradation of the newly synthesized metastatic repressor KAI1 occurring in cells with elevated concentration of the E3 ubiquitin ligase GP78 [54].

Cumulating data hint at the important role for maintenance of ER homeostasis played by the post-translational regulation of ERAD factors content in the ER lumen (reviewed in [55]). This regulation has been named ERAD tuning [55] and consists in the rapid and selective removal from the ER lumen of ERAD regulators. Unlike conventional folding chaperones and enzymes, several ERAD regulators such as ERManI [56,57], EDEM1 [45,58,59], OS-9 [45], XTP3-B [60], HERP [61,62] and SEL1L [63] are in fact rapidly removed from the ER lumen in unstressed cells. Some of them are degraded by the proteasome [61–63]; others, such as the ERManI [56,57], EDEM1 [45,58,59] and OS-9 [45] are degraded by endo-lysosomal enzymes.

**Figure 2.**
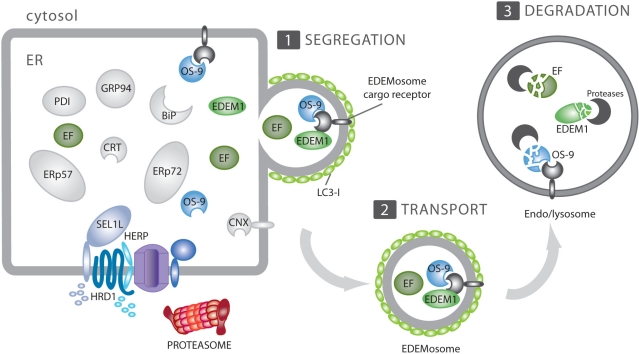
ERAD tuning. Many ERAD regulators are short-lived proteins at steady state. EDEM1 and OS-9 are canonical ERAD tuning substrates. Their selective removal from the ER lumen can be subdivided in three steps. (1) Association with an elusive membrane receptor allows segregation of EDEM1, OS-9 and possibly other ERAD factors (EF) from conventional, long-lived ER-resident chaperones (in grey); (2) The ERAD regulators exit the ER in small, LC3-I-coated vesicles, the EDEMosomes; (3) EDEMosomes deliver their content to endo-lysosomal compartments for disposal.

In 2007, Juergen Roth’s group revealed that EDEM1 is selectively released from the ER in vesicles [64]. More recently, we have shown that these carriers, which we have named EDEMosomes and display LC3/Atg8 at their limiting membrane, remove EDEM1 and other ERAD factors such as OS-9 from the ER lumen and transport them to endo-lysosomes for disposal (Figure 2) [45,58]. LC3 is a cytosolic ubiquitin-like protein that plays a crucial regulatory role in macroautophagy. Upon activation of this catabolic process that targets cellular components to lysosomes for degradation [65,66], LC3-I is converted into LC3-II by covalent conjugation to the membrane lipid phosphatidylethanolamine [67,68]. The covalent association of LC3-II to lipid bilayers appears to be essential to promote the elongation of the autophagosome membrane [69–71]. Interestingly, and unlike autophagosomes, the LC3-positive EDEMosomes are not decorated with ectopically expressed GFP-LC3 and do not contain LC3-II [58]. Rather, LC3-I is non-covalently associated to their limiting membrane and therefore this protein can be removed by carbonate extraction [59]. These results led us to propose an unconventional, autophagy-independent use of LC3 for the vesicle-mediated removal of short-living chaperones from the ER lumen [58].

Significantly, the DMVs in cells infected with MHV or SARS-CoV share several analogies with the EDEMosomes since they also derive from the ER and display LC3-I, but not ectopically expressed GFP-LC3 or LC3-II, at their limiting membranes [31,45,72]. In agreement with these results, a non-lipidable form of LC3 associates with the MHV-induced DMVs [45]. The finding that in MHV-infected cells the turnover of EDEM1 and OS-9 is essentially stopped and that these two proteins accumulate in the DMVs (they actually are the only cellular factors that distinctly co-localize with MHV-induced DMVs in immuno-fluorescence analyses [45]) led us to propose that the mechanism regulating the formation of the EDEMosomes is co-opted by CoV to ensure their efficient replication [45]. In keeping with this assumption, as previously reported for ERAD tuning [58], an autophagy-independent function of LC3 supports CoV infection. In fact, while the macroautophagy machinery regulating the covalent association of LC3 to the autophagosomal membranes is dispensable for infection as demonstrated by normal replication of MHV in *atg7^−/−^* mouse embryonic fibroblasts (MEFs), the reduction of the intracellular levels of LC3 by siRNA efficiently interferes with MHV replication [45]. Interestingly, when endogenous LC3 is replaced with a non-lipidable form of this protein, which cannot sustain autophagy, MHV infection is restored further supporting the notion of an unconventional use of LC3 in CoV replication.

These data explain previous observations. Originally, it was reported that the *ATG5* gene is essential for MHV replication in MEFs [73]. Careful reassessment of these findings in low passage *atg5^−/−^* MEFs and bone marrow derived macrophages lacking *ATG5* by virtue of a Cre recombinase mediated gene deletion, revealed that an intact autophagy pathway is not required for MHV life cycle [74]. In addition, these data also reconcile previous contrasting reports about LC3 association with CoV-induced DMVs. The works describing a co-localization were analyzing endogenous LC3, while those affirming the contrary used ectopically expressed GFP-LC3 [72–77].

## 5. Unanswered Questions

The rapid disposal of ERAD factors through the ERAD tuning and the hijacking of the ERAD tuning pathway by CoV are recent discoveries [45]. As a result, numerous questions still remain open. One scenario is that CoV anchor their replication and transcription complexes to the membranes of either EDEMosomes or “modified” EDEMosomes, whose fusion with a degradative endo-lysosomal compartment would be inhibited as a consequence of the infection. This would explain the defective EDEM1 and OS-9 turnover observed in infected cells and the enrichment of these two ERAD factors in the DMVs [45]. However, the molecular principles of the biogenesis of the EDEMosomes and DMVs are poorly understood (see above, [55,58,78,79]). In particular, the role of LC3-I in the formation of both EDEMosomes and CoV-induced DMVs remains unknown. One speculative idea is that LC3-I acts as a vesicle coat protein [79]. In such a scenario and similar to other vesicular transport pathways, one or more still elusive EDEMosome cargo receptors would bind EDEM1 and OS-9 in the ER lumen to segregate these short living ERAD factors from conventional and long-living molecular chaperones and folding enzymes. The cytosolic domain of this putative cargo receptor would then recruit cytosolic LC3-I. This latter step will be the key event required for the coat-driven formation of a carrier vesicle. Thus, one possible way for CoV to exploit the ERAD tuning machinery for generating their replicative DMVs would be to hijack one of the EDEMosome cargo receptors, perhaps by using one or more of their transmembrane non-structural proteins (*i.e.*, nsp3, nsp4 and/or nsp6). Alternatively, these nsp’s could act more directly by recruiting LC3-I and other vesicle coating factors. The first option contemplates that EDEM1 and OS-9 end up in the DMVs through their association with the EDEMosome cargo receptor. The second does not explain the peculiar distribution of these two chaperones in the MHV-induced DMVs, but it could be consistent with a model claiming that CoV may actively sequester EDEMosome cargo proteins such as EDEM1 and OS-9 into the DMVs in order to weaken the ERAD capacity in the ER lumen of the host cell. At the peak of its replication, CoV induce ER stress due to a sustained high production of viral components [80–83], including the 3 integral membrane nsps and the 3 structural membrane proteins that are initially inserted in the ER lipid bilayer. One of the consequences of the induction of ER stress is the enhancement of ERAD. As this would hamper CoV replication by degrading viral products, sequestering EDEM1 and OS-9, two positive regulators of the ERAD process, could limit this cellular response that would interfere with viral replication.

LC3 could also play a role in ERAD tuning and/or viral replication by linking EDEMosomes and the CoV-induced DMVs to the microtubule network, a notion suggested by the original full-length name of this protein, *i.e.*, microtubule-associated protein 1 light chain 3 (MAP1-LC3). The initial studies, revealed in fact that LC3 belongs to a family of microtubule-associated proteins and that it interacts with MAP1A or MAP1B to form a complex that binds and modulates the shape of microtubules [84–86]. Autophagosomes are mostly formed randomly at the periphery of the cell and redistribute in a microtubule-dependent way at the perinuclear region around the microtubule-organizing center (MTOC) where the majority of late endosomes and lysosomes are concentrated [87–90]. Recently, one of the molecular bases that could regulate this trafficking event has been revealed by showing that the N-terminus of LC3 interacts with FYCO1 (FYVE and coiled-coil [CC] domain containing 1), which in turn could interact with kinesin(s) [91]. Depletion of FYCO1 or antibodies against the N-terminus of LC3 blocks the subcellular redistribution of autophagosomes after completion [87,91]. Similarly, LC3 could link the EDEMosomes and the CoV-induced DMVs to the microtubule network. Interestingly, the MHV replicative structures, when visualized by using a GFP-tagged version of nsp2, were shown to be transported along microtubules. Upon disruption of this cytoskeletal scaffold, these structures remain dispersed throughout the cytoplasm and fail to concentrate to the perinuclear region [92]. Connecting CoV-induced DMVs with microtubules, however, cannot be the only function of this protein during an infection because while microtubules are dispensable for MHV replication in culture cells [92], depletion of LC3 affects MHV replication [45].

Whatever scenario holds true, it remains unclear how the DMVs are shaped from the single-membrane EDEMosome vesicles [79]. Furthermore, as EDEMosomes are probably derived from the smooth ER, it is not yet clear how to reconcile this with the observation that the surface of DMVs induced in SARS-CoV-infected cells is decorated with ribosomes [31], although ribosomes have not been observed on MHV-induced DMVs [33,45,79]. In addition, it remains to be solved how the alleged subversion of EDEMosomes by CoV results in the formation of a reticulovesicular network as observed in SARS-CoV infected cells [31].

## 6. Perspectives

Viruses are dependent on the host cell for virtually every step of the infection cycle and are able to subvert cellular processes to their own advantage. They can also be regarded as unique tools in cell biology research and have been used in the past decades to characterize a vast array of cellular machineries and pathways. Studying CoV replication and the biogenesis of the coronaviral replicative structures is therefore expected to increase our knowledge about the (re)shaping of cellular membranes and the formation of EDEMosomes, while the opposite also holds true. In addition, insight into virus-host interactions not only provides information into the cellular pathways hijacked by viruses, but also opens new avenues for therapeutic intervention. The absence of efficient anti-viral therapies emphasizes the necessity to further study and understand in detail the molecular mechanisms that regulate the CoV life cycle, including the role of LC3 in virus replication. This is desirable, as CoV are not only pathogens of veterinary importance, but a threat to mankind as well, as revealed by the emergence of the SARS-CoV. Furthermore, targeting of host rather than of viral proteins may, at least in some cases, be advantageous as it is less likely to result in viral escape mutants, as in contrast to the viral proteins, cellular factors are not prone to rapid mutations.
